# Interrelations of Intelligence and Social-Adaptive Skills in Adolescents with Multiple Developmental Disorders: A Pilot Study

**DOI:** 10.1192/j.eurpsy.2024.952

**Published:** 2024-08-27

**Authors:** E. Shvedovskii, N. Maltseva, D. Melnikova, S. Dronova, A. Bitova

**Affiliations:** ^1^ Mental Health Research Center; ^2^ Moscow State University of Psychology & Education; ^3^Regional non-profit social organization “Center for Curative Pedagogics, Moscow, Russian Federation

## Abstract

**Introduction:**

The relationship between social adaptation and intelligence in adolescents with developmental disorders varies depending on various psycho-social factors. Adolescence is marked by pubertal changes in mental and physical development. Previous research has revealed a moderate correlation between intelligence and various groups of adaptive skills in adolescents with Down syndrome. However, studies involving adolescents with multiple developmental disorders are relatively scarce in the existing literature

**Objectives:**

Determine the distribution of intelligence among adolescents with severe multiple disabilities; Identify the connection between intelligence and the level of adaptation in this group; explore the connection between intelligence and independence skills in the subjects.

**Methods:**

The study included 11 adolescent participants enrolled in a comprehensive social skills development intervention program at the Center for Curative Pedagogics: 5 girls and 6 boys, mean age - 14,0 yrs. Age st.dev: 24,3 and 18.4. ICD-10 DS of participants were: F48.xx, F70.xx, F80.xx, F84.xx, G40.xx, G80.xx, Q74.xx, and Q90.xx.

Following tools were used: Leiter-3 scales (LIQ), Vineland-3 Adaptive Behavior Scales (VSS); Perkins I.C.A.N. independence Scales (ICAN).

**Results:**

Selected variables including were tested with the Shapiro-Wilk test. p-values of the SW test indicated that data were not distributed normally: LIQ (w=0.953, p=0.685); VSS (w=0.964, 0.821); ICAN (w=0.877;p=0.095).

For the identification of the connections between the intelligence (LIQ) and adaptive functioning (VSS) we used r-Spearman criteria. These parameters showed significant monotonic relationship (rs = 0.961, p<0.001). Mean IQ level of the sample is characterized as mildly impaired (mean = 62.9). The adaptive and the independence skills level of the sample are also far below the low normative results (57.1 and 48.7 respectively). Images 1,2 and 3 shows the distribution of the data. The correlation between IQ (LIQ) and independence skills (ICAN) is not significant (rs = 0.671, p<0.024), as well as the correlation between adaptive and independence skills (rs = 0.733, p<0.010).

**Conclusions:**

We made an exploratory study of the adolescent participants of the comprehensive social skills development intervention program at the Center for Curative Pedagogics. Results show that non-verbal intelligence of the participants shows strong connection to the adaptive skills, but not to the independence skills. Sample size is very small, which is explained by the specifics of the intervention. Further research should be focused on the increasing sample and the expanding analysis parameters, such as social and family history, intervention details and the additional variables of the existing measurements.

**Disclosure of Interest:**

None Declared

